# High Performance Graphene Oxide Based Rubber Composites

**DOI:** 10.1038/srep02508

**Published:** 2013-08-26

**Authors:** Yingyan Mao, Shipeng Wen, Yulong Chen, Fazhong Zhang, Pierre Panine, Tung W. Chan, Liqun Zhang, Yongri Liang, Li Liu

**Affiliations:** 1 State Key Laboratory of Chemical Resource Engineering, Beijing University of Chemical Technology, Beijing. 100029, China; 2 State Key Laboratory of Organic-Inorganic Composites, Beijing University of Chemical Technology, Beijing. 100029, China; 3 Beijing National Laboratory for Molecular Sciences, Joint Laboratory of Polymer Science and Materials, Institute of Chemistry, Chinese Academy of Sciences, Beijing 100190, China; 4 Xenocs SA, 19 Rue François Blumet, 38360 Sassenage, France; 5 Department of Materials Science and Engineering, Virginia Polytechnic Institute and State University, Blacksburg, VA 24061, USA

## Abstract

In this paper, graphene oxide/styrene-butadiene rubber (GO/SBR) composites with complete exfoliation of GO sheets were prepared by aqueous-phase mixing of GO colloid with SBR latex and a small loading of butadiene-styrene-vinyl-pyridine rubber (VPR) latex, followed by their co-coagulation. During co-coagulation, VPR not only plays a key role in the prevention of aggregation of GO sheets but also acts as an interface-bridge between GO and SBR. The results demonstrated that the mechanical properties of the GO/SBR composite with 2.0 vol.% GO is comparable with those of the SBR composite reinforced with 13.1 vol.% of carbon black (CB), with a low mass density and a good gas barrier ability to boot. The present work also showed that GO-silica/SBR composite exhibited outstanding wear resistance and low-rolling resistance which make GO-silica/SBR very competitive for the green tire application, opening up enormous opportunities to prepare high performance rubber composites for future engineering applications.

Rubbers, generally acknowledged as strategic materials, can be tailored by adding fillers to meet the demands in versatile industry applications. Carbon black (CB) as a carbon nano-material has been widely used in rubber reinforcement for over 150 years. Nowadays, CB faces severe challenges because it is derived from crude oil and generates excessive wastes and greenhouse gas emissions. In order to tackle this global issue, new oil-independent fillers have received increasing attentions for replacement of CB.

Recently, graphene oxide^1^,^2^,^3^,^4^,^5^,^6^ (GO) has emerged as a new layered carbon material with nano size effects and unique physical properties^7^,^8^,^9^, implying that GO is a potential material for replacing CB for reinforcement of rubbers. Furthermore, graphite as the precursor of GO is oil-independent and naturally abundant. For successful application of GO in rubber industries especially tire industry, two criterions should be fulfilled: (i) commodity rubbers are selected as a matrix, such as styrene-butadiene rubber (SBR) and natural rubber, because these two kinds of rubbers account for over 60 per cent of the total rubber market in the world and are mainly reinforced by CB; (ii) the fabrication method is large scale and environment-friendly. However, most current studies for GO based rubber composites are limited in specific rubber matrices and focused on electric, thermal and some other functional properties^10^,^11^,^12^,^13^. In fact, in rubber industry, the static and dynamic mechanical properties of rubber composites are more concerned.

To fabricate high performance GO/rubber composites, it is essential to homogenously disperse GO sheets into rubber matrix. However, conventional mechanical blending method cannot uniformly disperse the GO sheets in the rubber matrix (see Supplementary Information S10). Solution mixing^12^,^13^,^14^,^15^ has been demonstrated to be an effective way to obtain the desired dispersion, but the removal of organic solvents poses persistent problems such as added cost and potential environmental pollution for the practical implementation of the method.

In this work, we prepared GO/SBR composites by the aqueous-phase mixing of GO colloid containing completely exfoliated GO sheets with SBR latex and a small loading of butadiene-styrene-vinyl-pyridine rubber (VPR) latex, followed by the co-coagulation of these components. By adjusting the flocculation process, VPR not only plays a key role in the prevention of aggregation of GO sheets during co-coagulation but also acts as an interface-bridge between GO and SBR. The GO/SBR composites formed by this route exhibit unprecedented reinforcing efficiency of GO towards SBR. The GO/SBR composites with small GO loadings have superior mechanical characteristics comparable with those of CB-filled SBR composites at high CB volume fractions, and also show low air permeability and low mass density. In addition, our preparation method is water-mediated, green, and large scale, which is appropriate for preparing various GO-based rubber composites since GO sheets can form well-dispersed aqueous colloid in water^16^,^17^,^18^ and most rubbers can exist in latex form.

## Results

**Figure 1:**
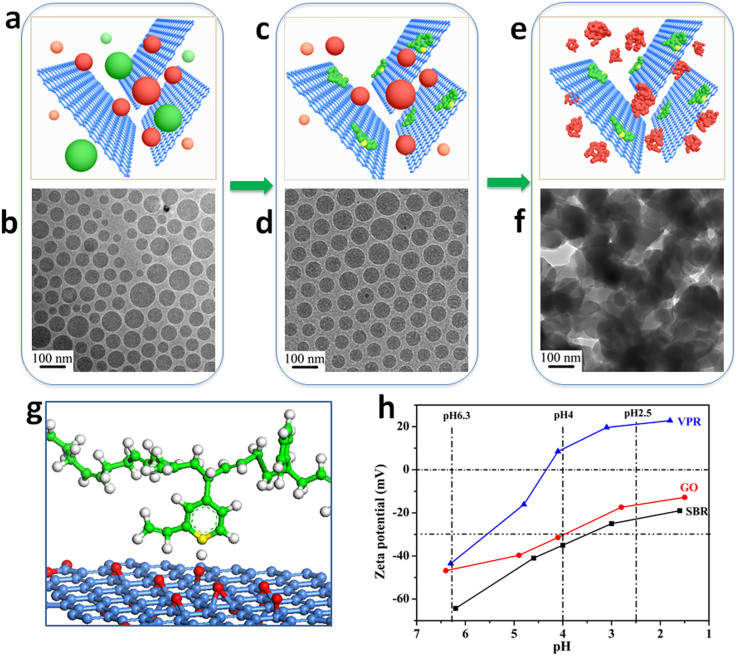
Fabrication process of GO/SBR composite. (a), (b), GO/VPR/SBR stable emulsion of 0.5 wt.% of GO sheets in an aqueous emulsion of 1 wt.% VPR and 9 wt.% SBR (1:9 w/w VPR-SBR) at pH 6.3. In the schematic representation (a), SBR and VPR colloidal particles are presented as red and green balls respectively. The cryo-TEM image of the corresponding GO/VPR/SBR stable emulsion is shown in (b). (c), (d), By adjusting pH value of mixture to 4.0 with sulfuric acid (H_2_SO_4_), the VPR colloidal particles are demulsified first, and the released VPR molecules are preferentially adsorbed onto the surfaces of the GO sheets because the acidified pyridine groups of VPR can interact with the ionized carboxylic acid and phenolic hydroxyl groups from the surfaces of the GO sheets. In the schematic representation (c), the released VPR molecules are represented as green random coil. The cryo-TEM image of the corresponding GO/VPR/SBR mixture is shown in (d). Ball-and-stick illustration of a model structure of the interaction between VPR and GO sheets is shown in (g). Yellow, red and gray are used to represent N, O and H atoms, respectively. (e), (f), When the pH is lower than 3.0, the SBR colloidal particles are further demulsified and then co-coagulated with VPR-modified GO sheets to form GO/SBR composite. In the schematic representation (e), the demulsified SBR molecules are represented as red random coil. The cryo-TEM image of the corresponding GO/VPR/SBR mixture is shown in (f). (h), Zeta potential of GO, SBR, and VPR as a function of pH, in aqueous dispersions at 0.5 wt.%, 5 wt.%, and 5 wt.% of GO, SBR, and VPR, respectively.

Figure 1 shows the fabrication process of GO/SBR composite. By aqueous-phase mixing of GO colloid with SBR and VPR latexes, we obtained GO/VPR/SBR mixture. The zeta potential of the mixture is −60 mv. As is well known from colloidal science, zeta potential values more negative than −30 mV are generally considered to represent sufficient mutual repulsion to ensure the dispersion stability^19^. It indicates the initial GO/VPR/SBR mixture exists as a stable state (Fig. 1a). A cryo-TEM image shows that colloidal particles with different sizes are individually dispersed in the mixture. However, no aggregates of GO sheets is observed, indicating that GO sheets may be isolated by rubber colloidal particles within the mixture dispersion (Fig. 1b).

By adjusting pH value of mixture to 4.0 with sulfuric acid (H_2_SO_4_), the size distribution of colloidal particles in the mixture becomes narrow compared with that at pH 6.3 and no GO aggregates can be found (Fig. 1d). It is supposed that the majority of the remained colloidal particles could be SBR but not VPR, which is verified by our zeta potential analysis (Fig. 1h). At pH 4.0, the zeta potential of SBR is still more negative than −30 mV. Meanwhile, the zeta potential of VPR has become positively charged, it is suggested that the VPR molecules are first released from colloidal particles, and then the N atoms in the pyridine of VPR molecules combine with H^+^ to form positively charged groups. Herein, the acidified VPR molecules will be strongly attracted to electronegative GO sheets (see Supplementary Information S4) to form VPR-modified GO sheets (Fig. 1g). In addition, the electrostatic repulsion between negative charged SBR colloidal particles ensured the VPR-modified GO sheets be separated uniformly in the mixture instead of aggregation (Fig. 1c).

When the pH approaches 2.5, the spherical morphology of SBR particles is destructed (Fig. 1f), indicating that SBR colloidal particles finally de-emulsificated and co-coagulated with the VPR-modified GO sheets to form the GO/SBR composite (Fig. 1e). This process made the exfoliated GO sheets were strongly combined by VPR chains, effectively preventing the aggregation of GO sheets^20^ and obtaining maximal amount of individual GO sheets in the GO/SBR composite, similar to those existed in the GO/VPR/SBR mixture.

In our experiments, we tried to coagulate the SBR latex and GO aqueous solution without VPR, but failed to get a GO/SBR composite with homogeneous dispersion of GO sheets. This result suggests that the electrostatic repulsion between the GO sheets is weaker than that between the GO sheets and SBR colloidal particles. Moreover, there is a strong steric repulsion between GO sheets and SBR colloidal particles. Therefore, GO sheets are strongly adsorbed by H^+^ ions (Fig. S9), and the SBR latex particles between the GO sheets are expelled, resulting in large GO aggregates in the final composite (Fig. S10).

**Figure 2:**
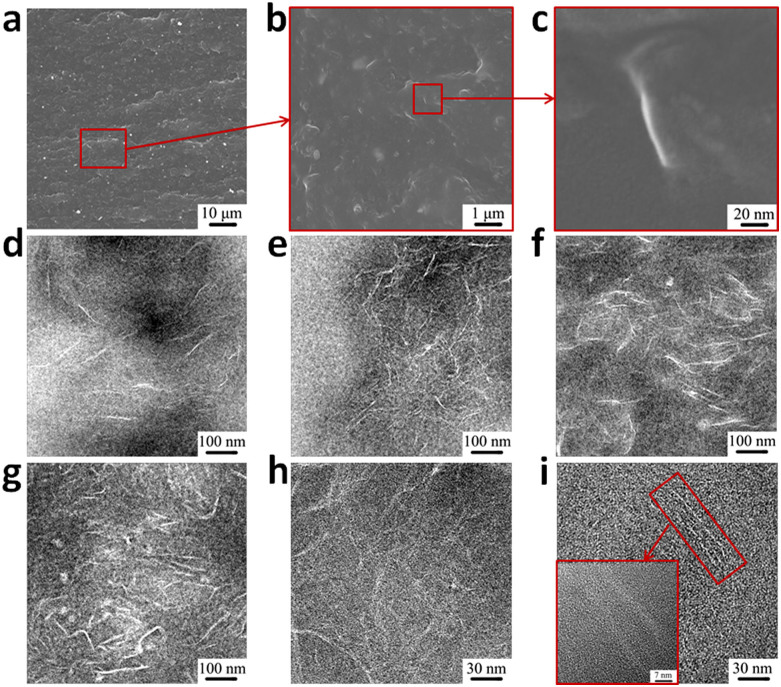
Morphology images of GO/SBR composite. (a–c), SEM images ((a), 1,000× magnification; (b), 20,000× magnification; (c), 200000× magnification) of tensile sections of GO/SBR composite with 2.0 vol.% of GO. (d–g), TEM images of microtomed SBR/GO composites revealing different morphologies of GO sheets, including crumpling and folding, at different concentrations (vol.%): (d), 0.2; (e), 0.4; (f), 1.2; (g), 2.0. (h–i), High-resolution phase-contrast images of different regions of microtomed GO/SBR composite sample (2.0 vol.% of GO) at different magnifications. These high-resolution images show individual sheets and/or layer-by-layer sandwich structures of GO.

The dispersion state of GO sheets in GO/SBR composites was observed by SEM and the images are shown in Fig. 2a–c. No GO aggregates are observed on the cross-section of the composite (see Fig. 2a). Some wrinkled GO sheets are pulled out of the SBR matrix, as pointed out by the arrows in Fig. 2c. The GO-based sheets in the rubber matrix are much thicker than the original GO sheets (Fig. S1b) as determined by AFM because their surfaces are coated with VPR rubber.

Transmission electron microscopy (TEM) was used to determine the dispersion structure of GO sheets. Extensive high-resolution phase-contrast images of GO/SBR composite at different GO loadings (Fig. 2d–g) show no evidence of multi-layer stacks. At a GO loading of 0.4 vol.%, the composite is entirely filled with the GO sheets, as shown in Fig. 2e, owing to the extremely large surface area of the sheets. In contrast to the typically flat GO sheets observed by AFM (Fig. S1b), the GO sheets in the GO/SBR composite are crumpled and folded. The structures of the GO sheets dispersed in the rubber matrix include both individual GO sheets (Fig. 2h) and “layer-by-layer” sandwich structures consisting of GO sheets and rubber (Fig. 2i).

**Figure 3:**
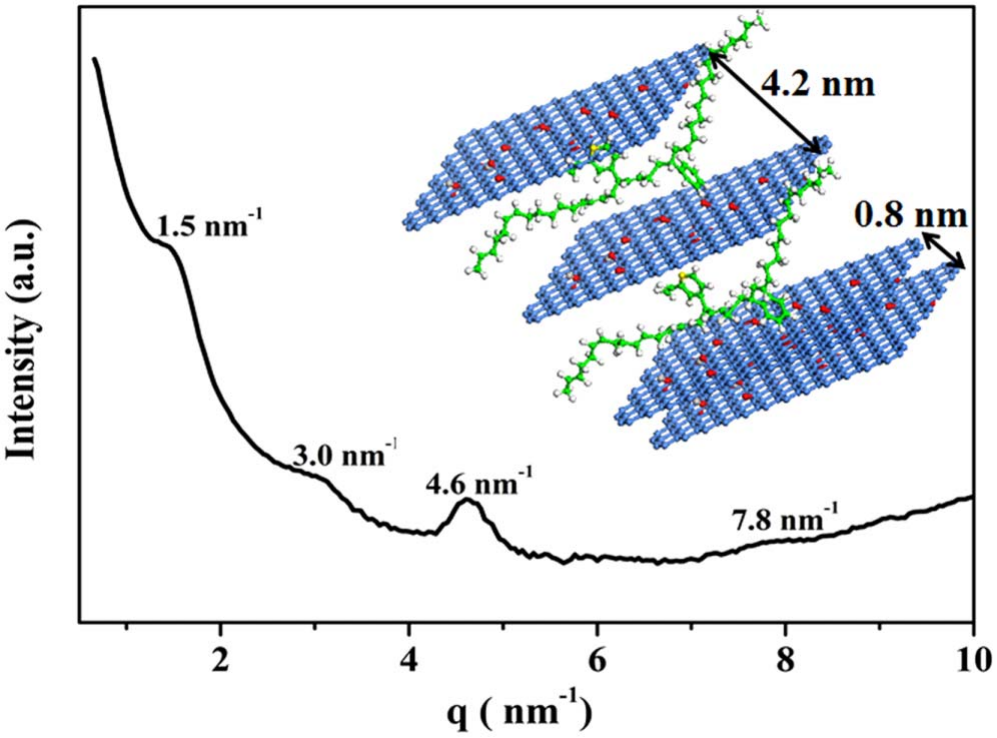
SWAXS pattern of GO/SBR composite. The three peaks at 1.5, 3.0, and 4.6 nm^−1^ indicate a typical high order lamellar structure with a long period of 4.2 nm. The weak and broad peak at 7.8 nm^−1^ indicates a distance of 0.8 nm. The inset shows the interlayer distance of the sandwich structure of GO/SBR composite. In the schematic representation of “layer-by-layer” sandwich structures consisting of GO sheets and rubber, the GO sheets are presented in blue lattice structure, the rubber between GO sheets are presented in green chains.

Small-angle/wide-angle X-ray scattering (SWAXS) was performed to further verify the structures of GO sheets dispersed in the SBR matrix in nano-scale. The SWAXS profile of the composite shows four scattering peaks at 1.5, 3.0, 4.6, and 7.8 nm^−1^ (Fig. 3). The three peaks at 1.5, 3.0, and 4.6 nm^−1^ indicate a typical high order lamellar structure with a long period of 4.2 nm, which is 4.5 times longer than the layers distance (d = 0.96 nm) in pure GO (Fig. S2). The weak and broad peak at 7.8 nm^−1^ indicates a distance of 0.8 nm, which is very close to the interlayer distance of GO. These results indicate that different microstructures were formed in the GO/SBR composite. Most GO sheets were dispersed in the rubber matrix as individual sheets. However, owing to the strong electric attraction and nano-scaled effect between the GO sheets, the aggregation of GO sheets is kinetically favored during the co-coagulation process. It is worth pointing out that only sandwich structures (see inset of Fig. 3), instead of aggregates, were formed in the GO/SBR composite, evidence that well-dispersed individual GO sheets in aqueous solution can be first modified with VPR chains released from latex particles to form a unique GO/VPR meso-structure. This GO/VPR meso-structure can play a key role in protecting the original structures of the highly dispersed individual GO sheets in the aqueous solution from re-aggregation during the co-coagulation process.

## Discussion

**Figure 4:**
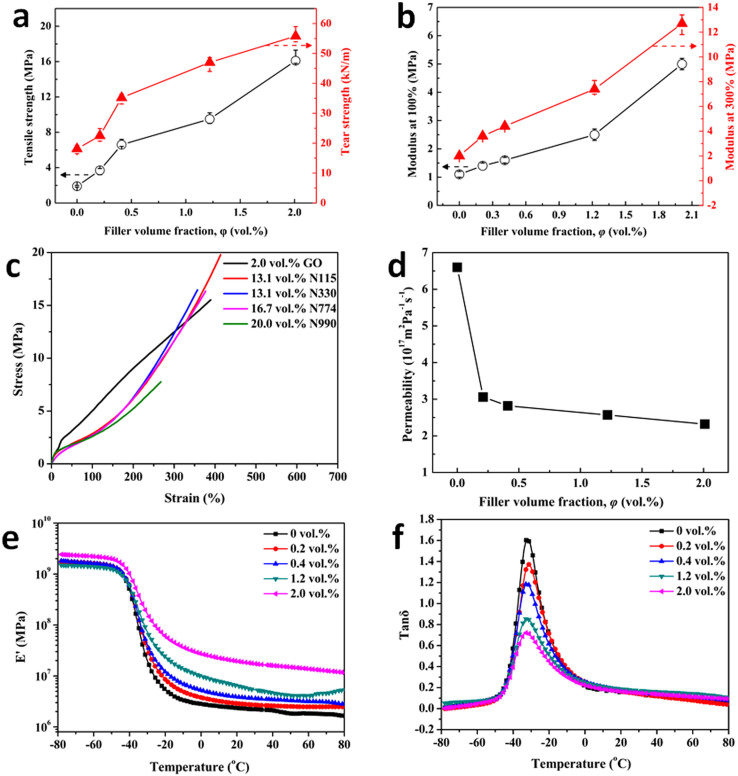
Properties of GO/SBR composite at different GO volume fractions. (a), (b), Tensile strength and tear strength (a), modulus at 100% and 300% elongation (b) of GO/SBR composite at different GO volume fractions. Symbols denote experimental values, and experimental errors were derived from measurements acquired from different regions of the same sample. (c), Stress-strain curves of SBR composites containing various fillers at different filler volume fractions. (d), Gas permeability of GO/SBR composite as a function of GO loading. (e), Storage modulus versus temperature for neat SBR and SBR/GO composite at different GO contents. (f), Tan delta versus temperature for neat SBR and SBR/GO composite at different GO contents.

The GO dispersion in composite and the interface interaction are the two main factors to determine the reinforcing efficiency of GO in the composite^21^. The typical stress-strain curves and the mechanical properties of GO/SBR composites with various GO loadings from 0 to 2.0 vol.% are summarized in Fig. S11 and Table S1 (Supplementary Information S6), respectively. The tensile strength and tear strength of the composite increased by as much as 700% and 208%, respectively, over those of SBR after the addition of 2.0 vol.% of GO (Fig. 4a). With increasing GO content, the modulus at 100% elongation (M100) and the modulus at 300% elongation (M300) of the composite increase (Fig. 4b), but with no decrease in elongation at break (Fig. S11).

The excellent reinforcement by GO sheets can be attributed to two reasons. First, as for fully-exfoliated GO sheets, GO has a large specific area of about 2600 m^2^g^−1^. Therefore the GO provides nearly 1.3 × 10^4^ m^2^ of sheet surface area in GO/SBR (2.0 vol.%) composite theoretically. Accordingly, there is a large contact area between the GO sheets and the rubber matrix in the GO/SBR composite, although some stacks of multiple GO sheets and the nanoscale corrugation of the GO sheets^22^ in the composite lead to a lower surface area than the theoretical value. Second, there is a glassy layer in the vicinity of the GO sheets as a result of the ionic interactions between the GO sheets and VPR, the chains of which can co-crosslink with the SBR chains. The rubber chains in the glassy layer will slip along the GO sheets and form stretched and parallel-arraying straight chains^23^ under external stresses. After the orientation of chains, the stresses can be uniformly shared by the oriented chains to avoid stress concentrations^24^. Meanwhile, the glassy layer also acts as a mediator to facilitate the stress transfer from the matrix to the GO sheets.

As shown in Fig. 4c, the tensile strength of the composite filled with 2.0 vol.% of GO is twice as high as that of the rubber composite filled with 20.0 vol.% of the CB N990 and almost the same as that of the rubber composite reinforced by 13.1 vol.% of the CB N115 (average particle size of 30 nm, most effective reinforcing filler for rubber in the rubber industry). The experimental results demonstrated that the reinforcement efficiency of GO fillers is higher than that of CB, and this improvement comes with a low mass density of the composite. Moreover, any eventual replacement of CB by GO will have epoch-making significance in the oil-dependent rubber industry. According to data from the International Rubber Study Group, the global consumption of CB in 2010 was 10.1 million tons, and this number is expected to reach 11.5 million tons by the year 2015. This amount of CB will consume 25.3–28.8 million tons of crude oil^25^. In contrast, GO, which is oil-independent, can be readily prepared from different types of graphite, all inexpensive.

The fully-exfoliated GO and its good dispersion in rubber also makes it as a good candidate for gas-resistant materials. A “tortuous path” among the fillers that retard the progress of the gas molecules through the matrix should be formed in the rubber composite to obtain low gas permeability^26^. As the gas-permeability vs. GO loading results presented in Fig. 4d, the percolation loading occurs at a GO loading of only 0.4 vol.% in GO/SBR composites. This percolation threshold is about 40 times lower than that reported for clay-based composites^27^. According to the Lewis-Nielsen model^28^ on gas permeability, the thickness of an individual GO-based sheet dispersed in the GO/SBR composite with 2.0 vol.% of GO was predicted to be 1.47 nm (see Supplementary S8 for detailed calculations). This also verified that the exfoliated structure of the GO sheets in the SBR matrix. The promising barrier property of GO/SBR composite is very competitive for such applications as tire inner liner and rubber balloon.

Dynamic mechanical properties were therefore investigated in this work since many engineering rubbers are operated under dynamic loading. The storage modulus (E′) and the loss factor (tanδ) of neat SBR and GO/SBR composites versus temperature are depicted in Fig. 4e and Fig. 4f, respectively. The storage modulus reflecting the elastic response of a rubber, is a measure of the recoverable strain energy in the rubber. As shown in Fig. 4e, the storage modulus of the SBR/GO composite increases with the GO content increasing, throughout the range of temperatures investigated, indicating that the elastic response of neat SBR towards deformation is strongly influenced by the presence of nanodispersed GO sheets.

Tanδ of rubber composites represents the ratio of the energy loss to the energy stored. In Fig. 4f, a glass transition region (from −48°C to −3°C) is observed in the curves of tanδ versus temperature. Tanδ at a given temperature decreases with increasing GO content. The tanδ peak decreases with increasing GO loading, indicating that there was a reduction in heat buildup and damping capability of SBR systems^29^ with GO loading increases.

**Figure 5:**
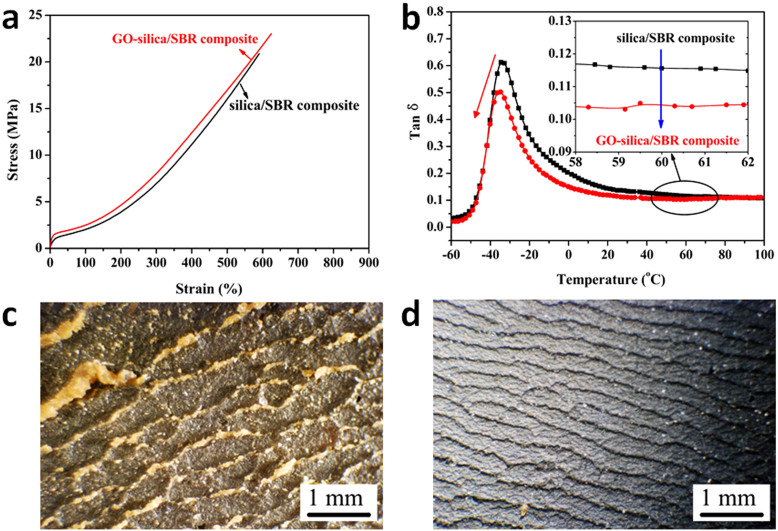
Properties of silica/SBR composite and GO-silica/SBR composite at total filler loading of 20.1 vol.%. (a), Stress-strain curves for silica/SBR composite (0 vol.% GO, 20.1 vol.% silica) and GO-silica/SBR composite (0.6 vol.% GO, 19.5 vol.% silica). (b), Mechanical loss factor (tanδ) as a function of temperature for silica/SBR composite and GO-silica/SBR composite. (c–d), Optical images (40× magnification) of Akron abrasion surface of silica/SBR composite (c) and GO-silica/SBR composite (d).

The GO/SBR composite shows great versatile application in rubber industry, especially in modifying silica-reinforced rubber composite which is currently used in preparing passenger tire with low rolling resistance. The “green” GO/SBR composite was mixed with silica/SBR composite to get GO-silica/SBR composite. The result shows that, in the same filler loading, GO-silica/SBR composite exhibits more outstanding mechanical properties than those of the silica/SBR composite (Fig. 5a). When the GO content is up to 0.6 vol.%, the tanδ at 60°C was about 10% lower than that of the silica/SBR composite (Fig. 5b). Generally, the rolling resistance of silica-reinforced rubber composite is about 10–35% lower than that of CB-reinforced rubber composite, indicating the great potential of GO/SBR composite for low rolling-resistance tire material. Moreover, a large amount of debris and deep wear patterns were found on the worn surface of silica/SBR composite (Fig. 5c), corresponding to a poor wear resistance (Akron wear 0.27 cm^3^/1.61 km). But for the GO-silica/SBR composite, the worn surface became smoother and showed much narrower wear patterns (Fig. 5d), corresponding to an improved wear resistance (Akron wear 0.19 cm^3^/1.61 km).

In summary, the present work demonstrated that GO sheets can be facially integrated into a rubber matrix by a new, versatile, and extremely simple approach based on latex technology. In this approach, a specific rubber-GO interaction and well-dispersed GO sheets in rubber matrix were obtained with the assistance of VPR to maximize the reinforcing efficiency. The results also verified that the resulting composite can be easily processed and exhibit excellent mechanical properties and gas barrier property. By mixing the “green” GO/SBR composite with silica/SBR composite, the GO-silica/SBR composite showed improved static and dynamic mechanical properties compared with those of silica/SBR composite in the same filler loading. The outstanding wear resistance and low-rolling resistance make GO-silica/SBR very competitive for the green tire application. This work might lead to future scalable production of GO-based rubber composites, and provides a new insight into the fabrication of high performance rubber composites for future engineering applications.

## Methods

### Preparation of composites

Graphite oxide was prepared from flake graphite (Fig. S1a) by a modified Hummers method^30^. Then a mild ultrasonic treatment of graphite oxide in water results in its exfoliation to form stable graphene oxide (GO) colloid. Proper ratio of the GO colloid containing 0.35 wt.% solid with 1 nm thick sheets (Fig. S1b), SBR latex containing 20.0 wt.% solid with 60 nm diameters (Fig. S1c), and VPR latex containing 5.0 wt.% solid with 70 nm diameters (Fig. S1d) were mixed by vigorous stirring for 30 min. The GO/VPR/SBR emulsion (Fig. S1e) was then co-coagulated by a 1.0 wt.% sulfuric acid solution. The coagulated composites (Fig. S1f) were washed with water until the pH of the filtered water reached 6–7 and then dried in an oven at 50°C for 24 hours. The dried composites were compounded with rubber ingredients on an open two-roll mill and subjected to compression in a standard mold at 150°C and 15 MPa for an optimum time, which was determined by a disc rheometer (Model 750, Beijing Huan Feng Mechanical Factory). The formulation of the GO/SBR composites was as follows: 90 g of SBR; 10 g of VPR; 0–5 g of GO; 5 g of zinc oxide; 2 g of stearic oxide; 3 g of antioxidant (4010NA); 1.4 g of accelerator CBS; 0.1 g of accelerator M; 2.8 g of sulfur. CB/rubber composites were prepared first by dispersing CB within rubber on an open two roll mill. After the rubber was melted for 1 min, the CB was slowly added. The total blending process took 10 min, and the mixing speed was maintained at 40 rpm/min. Subsequent processing was the same procedure as that of GO/SBR composites.

### Analytical measurements

Cryo-TEM observations were conducted with a PHILIPS Tecnai 20 (France) at 200 kV. Ultra-thin sections of composite samples for TEM observations were cut by a liquid-nitrogen-cooled microtome at −100°C, collected on a copper grid, and then observed on a JEM-3010 HR-TEM (JEOL, Japan) operated at 300 kV. A Hitachi S-4700 scanning electron microscopy (SEM) was used to examine the composites cross-section. Small-angle/wide-angle X-ray scattering (SWAXS) of the samples was performed on a Xenocs-3D SAXS (France). The permeation experiments with nitrogen were carried out with a gas permeability apparatus (pressure difference method). Tensile tests were performed on a CTM 4104 tensile tester (SANS, Shenzhen, China) at a crosshead speed of 500 mm/min at 25 ± 2°C. The storage modulus E′ and dynamic loss factor (tanδ) as a function of temperature were measured with a DMTA V dynamic mechanical thermal analyzer (Rheometrics Science Corp., USA) in the tensile mode at 10 Hz and heating rate 3°C/min.

## Author Contributions

Y.M. carried out the experimental work, including the preparation and some characterization of the composites. L.L. and Y.L. supervised the experimental research. P.P. carried out the SWAX characterization of the composite. S.W., Y.C., F.Z., L.Z. and T.C. all made contributions to the manuscript.

## Supplementary Material

Supplementary Information Supplementary information
