# Supplementary material for: High Performance Graphene Oxide Based Rubber Composites
Source: Sci Rep. 2013 Aug 26;3:2508. doi: 10.1038/srep02508 (PMC3752610; doi:10.1038/srep02508)
Supplement: Supplementary Information [file srep02508-s1.doc]

**High Performance Graphene Oxide Based Rubber Composites**

Yingyan Mao^1^, Shipeng Wen^1^, Yulong Chen^1^, Fazhong Zhang^1^, Pierre Panine^4^, Tung W. Chan^5^, Liqun Zhang^2^, Yongri Liang^3*^ and Li Liu^1*^

1. State Key Laboratory of Chemical Resource Engineering, Beijing University of Chemical Technology, Beijing, 100029, China;

2. State Key Laboratory of Organic-Inorganic Composites, Beijing University of Chemical Technology, Beijing, 100029, China;

3. Beijing National Laboratory for Molecular Sciences, Joint Laboratory of Polymer Science and Materials, Institute of Chemistry, Chinese Academy of Sciences, Beijing 100190, China;

4. Xenocs SA, 19 Rue François Blumet, 38360 Sassenage, France ;

5. Department of Materials Science and Engineering, Virginia Polytechnic Institute and State University, Blacksburg, VA 24061, USA.

**S1. Preparation of GO/SBR composite**


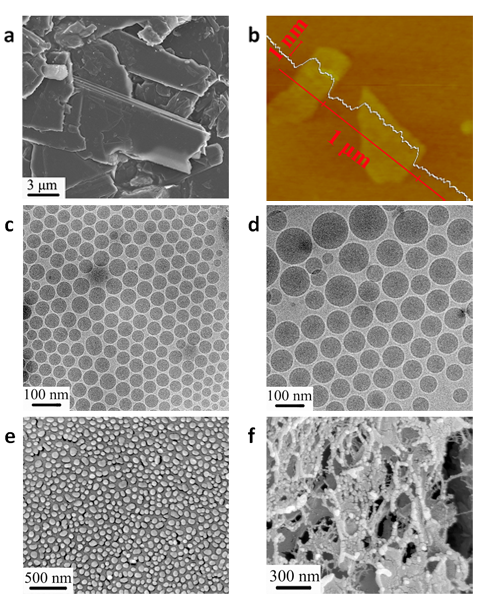


**Figure S1.** **Images of the samples obtained in each step. a,** SEM image of natural graphite. **b,** A tapping mode AFM topographic image and height profile of GO. **c,** Cryo-TEM image of 20 wt.% neat SBR latex. **d,** Cryo-TEM image of 5.0 wt.% neat VPR latex **e,** Cryo-SEM image of a 5:10:90 w/w/w GO/VPR/SBR aqueous mixture. **f,** Cryo-SEM image of GO/SBR composite (2.0 vol.% of GO).

**S2. Characterization of graphite oxide obtained by oxidation of graphite**

The XRD spectra patterns of graphite and graphite oxide are presented in Fig S2 to investigate the sheet structure. As shown in Fig. S2, a sharp peak appears at 2θ=26.5° for graphite, corresponding to a d-spacing of 0.34 nm. Compared with the diffraction peak of graphite, the peak of graphite oxide becomes broader and the peak value shifted to 2θ=9.2°, corresponding to a d-spacing of 0.96 nm, as a result of the intercalation of the functional groups into the interlayers of graphene sheets.


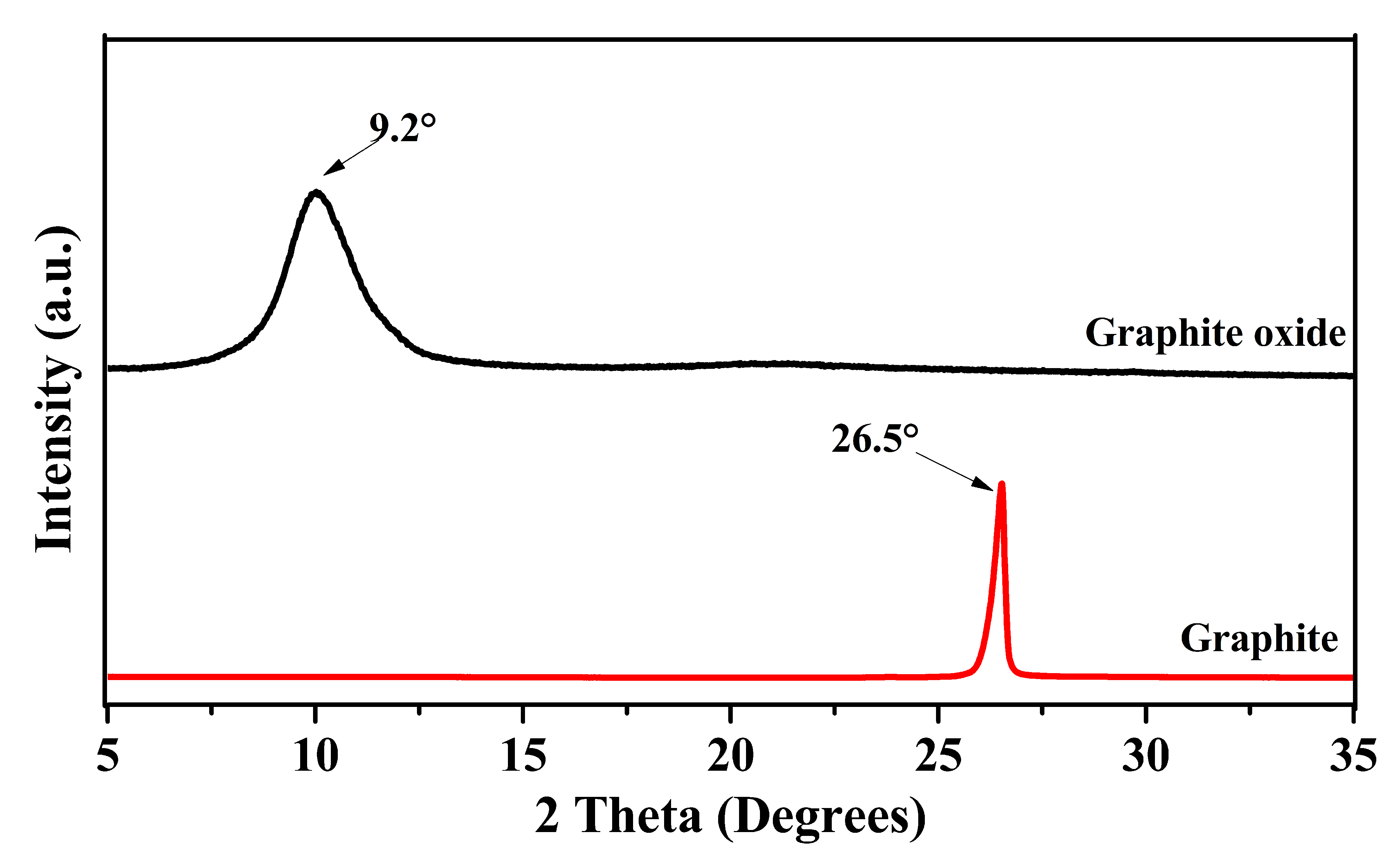


**Figure S2.** XRD spectra of graphite and graphite oxide

The FTIR was carried out to analyze the functional groups of GO. In the FTIR spectra of GO and graphite (Fig S3), the peaks at 3427, 1726, 1691, 1401, and 1044 cm^-1^ for GO are attributed to O-H stretching vibrations, C=O stretching vibrations, C=C stretching vibrations, O-H deformations, and C-O stretching vibrations, respectively. As a result of ionization of the carboxylic acid and phenolic hydroxyl groups on the GO sheets, the GO sheets are highly negatively charged when dispersed in water (the zeta potential of the GO aqueous dispersion was determined to be -46.8 mV at pH 6.4). Because of the electrostatic repulsion between GO sheets and the hydrophilicity of GO, the exfoliated GO can form stable aqueous colloids.


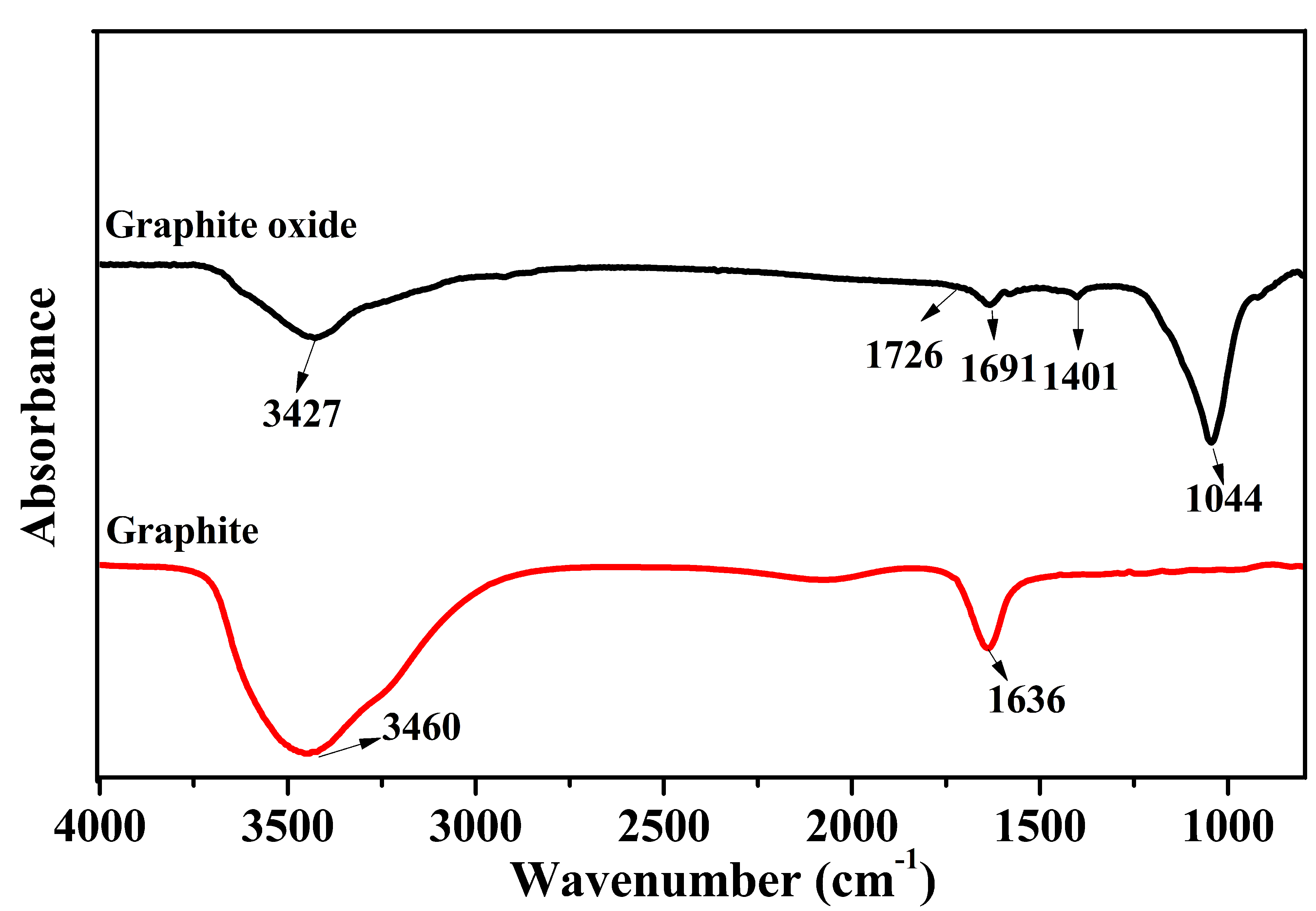


**Figure S3.** FTIR spectra of graphite and graphite oxide

**S3. Stability of SBR latex, VPR latex, GO colloid, and GO/VPR/SBR mixture**

Fig. S4 shows the particle or cell size distribution of GO aqueous, SBR latex, VPR latex and their mixture results measured by dynamic light scattering (DSL). It should be noted that the measured size of GO does not reflect the actual size because the measurement was based on the assumption that the particles are spherical. In addition, the radius of SBR and VPR latex particles measured by DSL are larger than that seen from cryo-TEM image (Fig. S1c-d), owing to the SBR and VPR latex particles contracted with cooling during the cryo-TEM observation. Nevertheless, it provides a means of determining dispersion stability. No aggregates were observed in GO/VPR/SBR mixture, indicating the uniform dispersion of GO sheets in GO/VPR/SBR mixture.


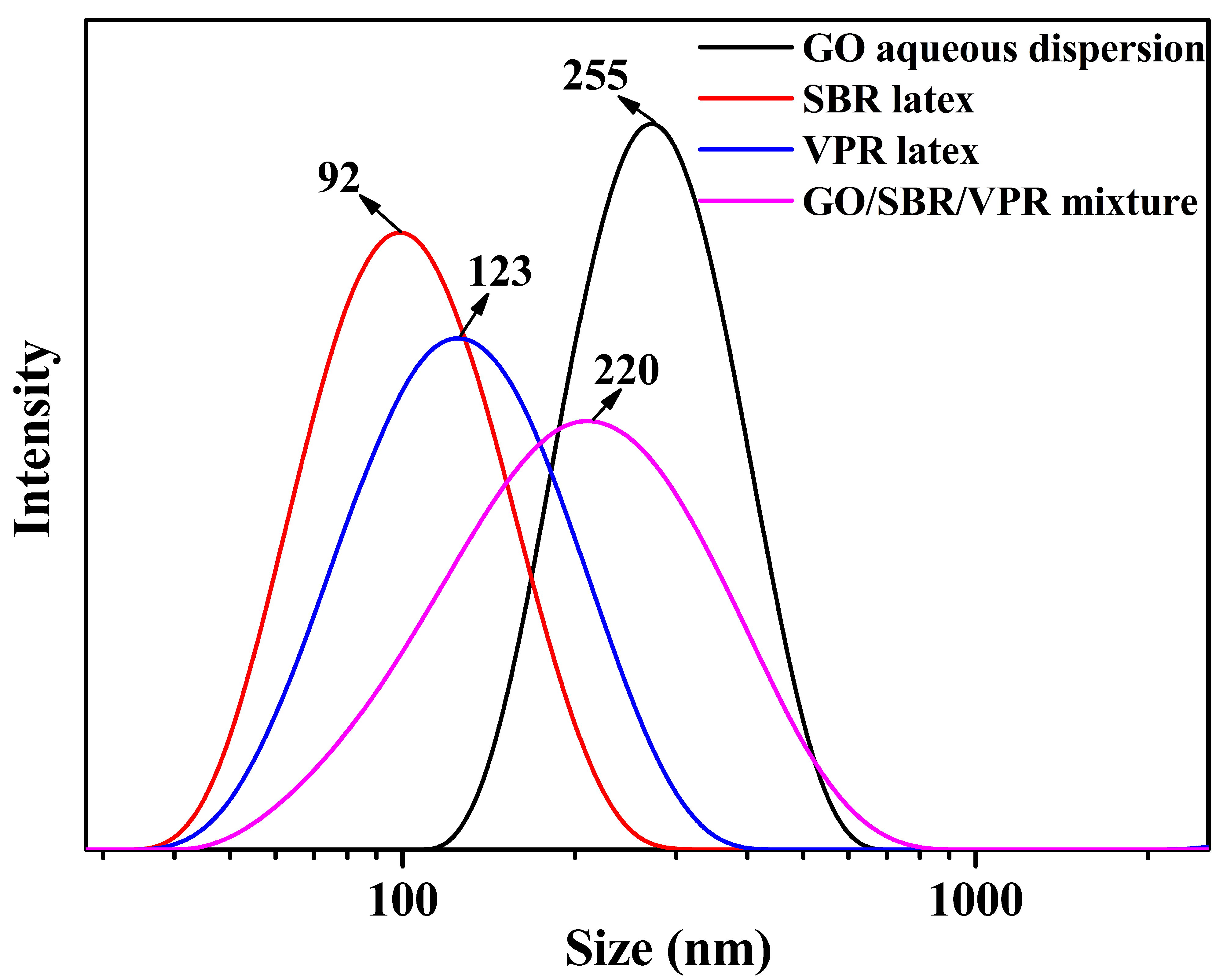


**Figure S4.** Size distributions of SBR latex, VPR latex, GO colloid, and

GO/VPR/SBR mixture with 2.0 vol.% GO

**S4 Interface interaction between GO and VPR**

VPR solution and SBR solution were first prepared by mixing coagulated VPR and coagulated SBR into toluene, respectively, and then poured into GO colloid to make GO/VPR and GO/SBR mixtures, respectively, at toluene/H_2_O (1/1, v/v). Since toluene and H_2_O are immiscible, phase separation occurred within H_2_O phase at the bottom and toluene phase at the top (Fig. S5). GO was completely dispersed in water because of its hydrophilicity, while the rubber matrix was readily dispersed in toluene. For the GO/VPR mixture, a transition layer at the interface was observed (Fig. S5a), an evidence of the interaction between GO and VPR at the interphase. For the GO/SBR system, a clear interface between the SBR toluene solution and GO aqueous suspension was observed (Fig. S5b).


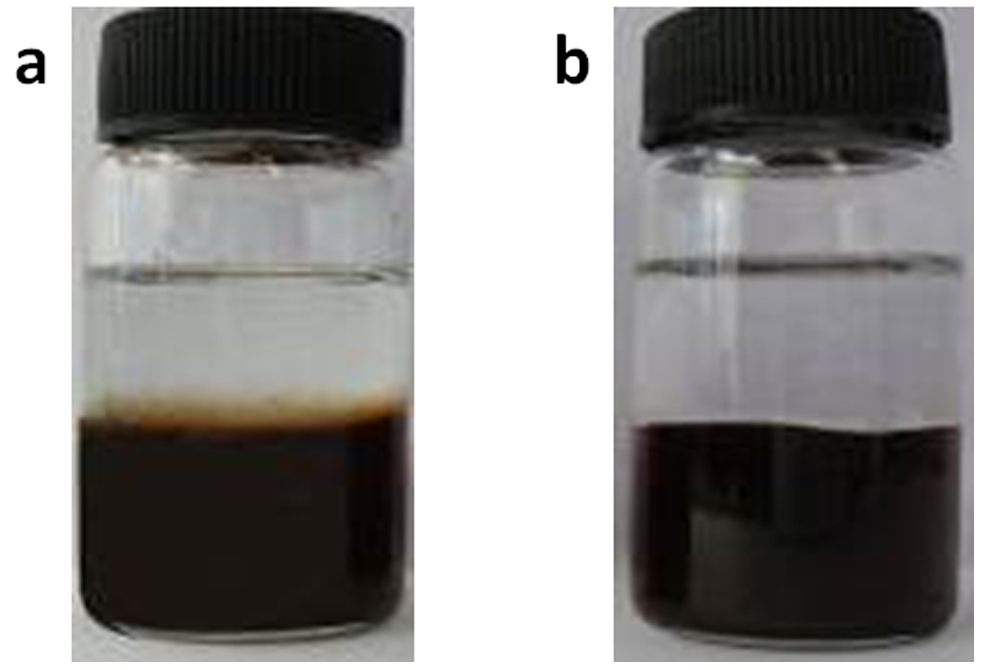


**Figure S5.** Photographs of (a) GO/VPR mixture and (b) GO/SBR mixture in toluene/H_2_O (1/1, v/v) solvent.

The XPS spectra of neat VPR and GO/VPR complex are shown in Fig. S6. The neat VPR is obtained from directly drying VPR latex. The GO/VPR complex is obtained from co-coagulation of VPR latex and GO colloid by H_2_SO_4_. The N 1s band of the neat VPR (Fig. S6a) was reconstructed into two peaks. The binding energies at 399.2 eV and 400.1 eV are assigned to nitrogen in the 2-vinyl pyridine unit of VPR. The two peaks at 399.2 eV and 400.1 eV shift to 399.5 eV and 400.4 eV respectively in the XPS spectrum of the GO/VPR complex (Fig. S6b), indicating that the electron-rich GO shares its electron cloud with the nitrogen nuclei. Furthermore, there is a new peak centered at 401.9 eV, which corresponds to protonated nitrogen, indicating ionic interactions between VPR molecules and GO sheets.


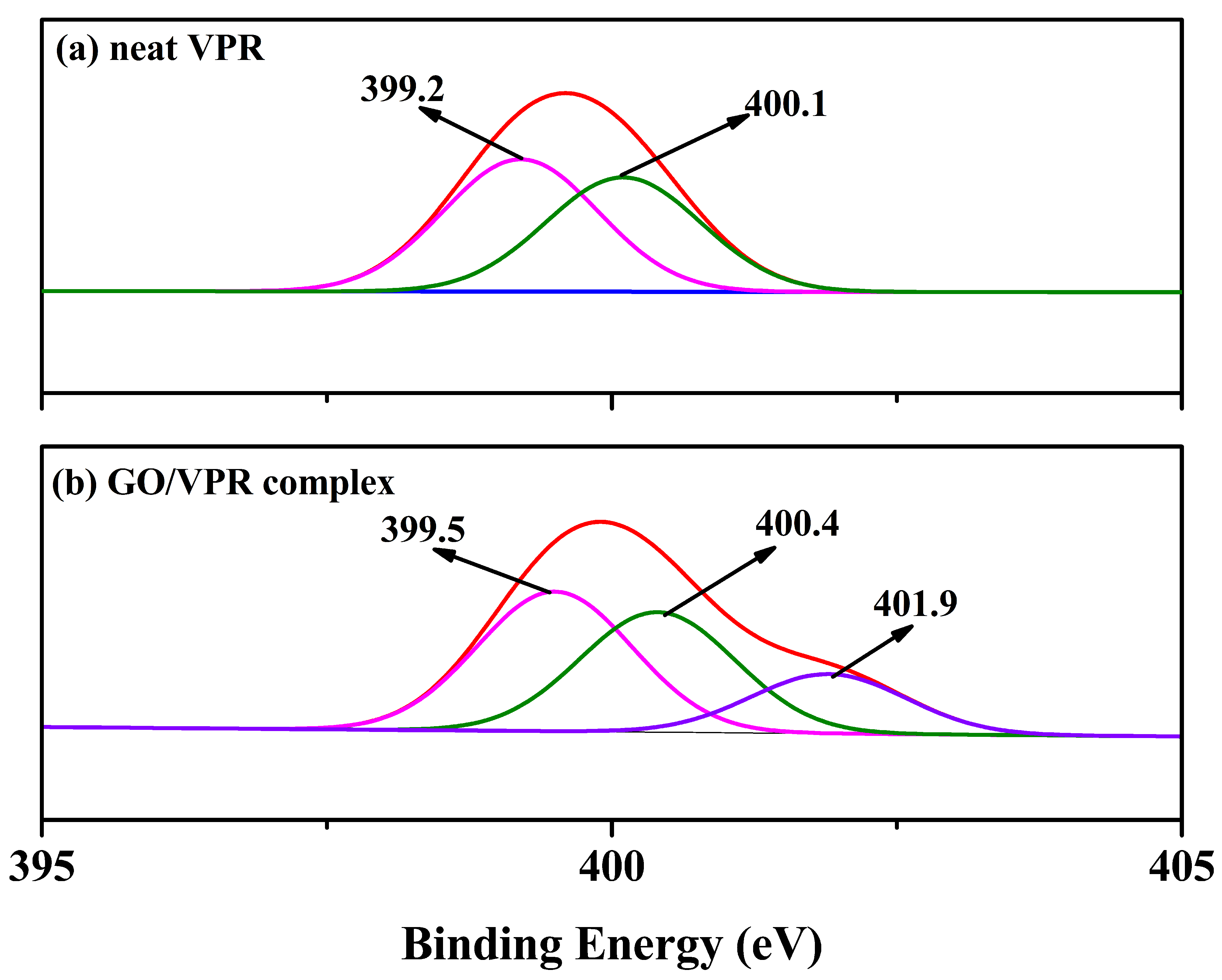


**Figure S6.** XPS N 1s core-level spectra of neat VPR and GO/VPR complex.

The FTIR spectra of neat VPR and GO/VPR complex are shown in Fig. S7. The peak of -C=N in the pyridine ring appears at 1590 cm^-1^ in the FTIR spectrum of neat VPR, while the same peak shifts to 1595 cm^-1^ in the spectrum of GO/VPR complex, another sign of the interaction between VPR and GO.


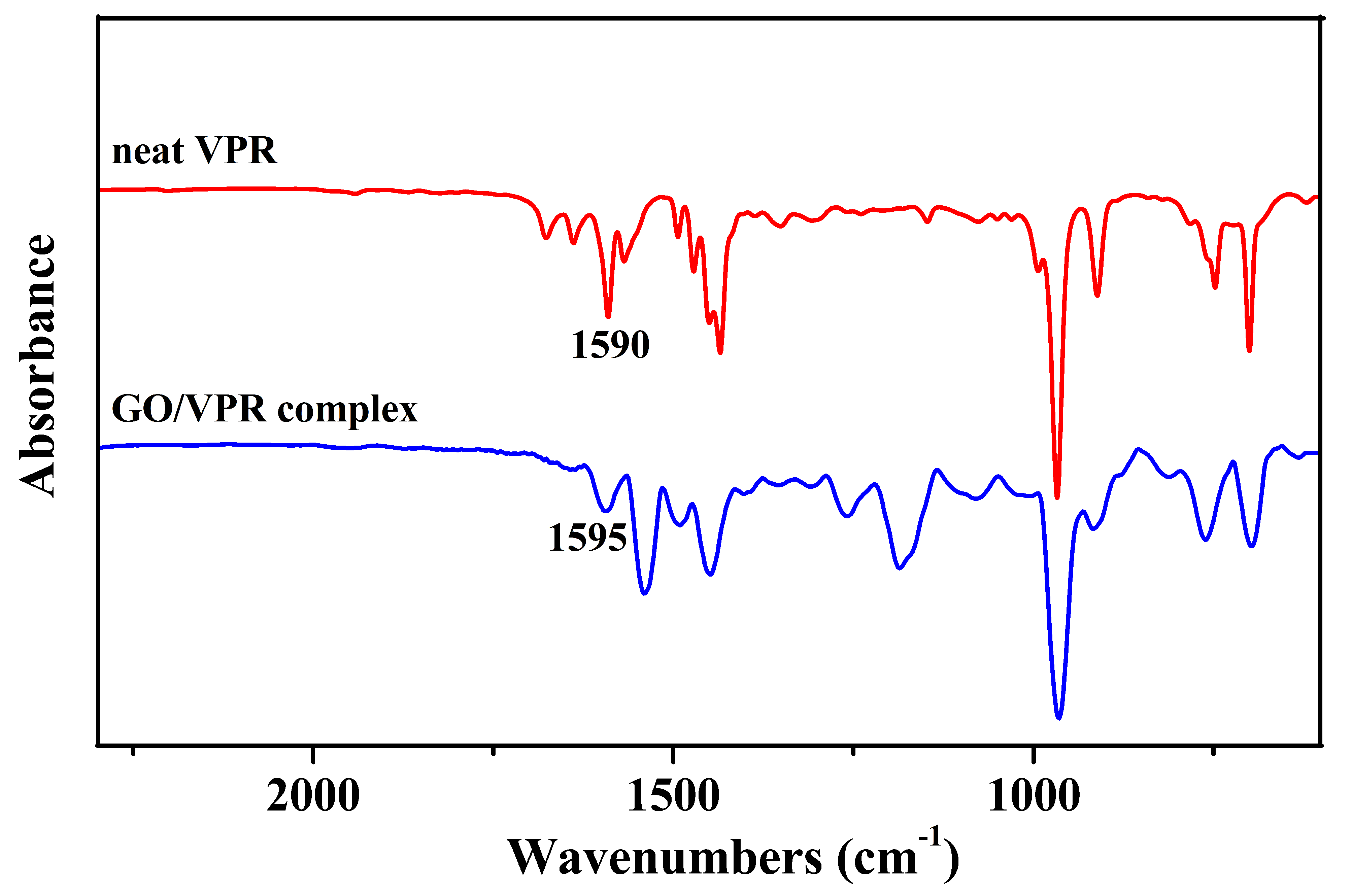


**Figure S7.** FTIR spectra of neat VPR and GO/VPR complex.

Solid state ^15^N NMR is applied to further prove the interaction between the GO sheets and VPR molecules. The spectra of gum VPR by direct drying and GO/VPR complex are shown in Fig. S8. The single peak of curve (a) in Fig. S8 indicates that the chemical shift of the N atom of the pyridine group attached by the VPR is 141.1 ppm. However, as shown in curve (b), the peak is shift from 141.1 ppm to 135.1 ppm in GO/VPR complex. This is obviously related to the change in the chemical environment of the N atom and is can only be ascribed to the combination of a N atom with H^+^ through the donation of its lone electron pair and the protonized pyridine group of VPR molecules interact with electronegative GO sheets through ionic bond.


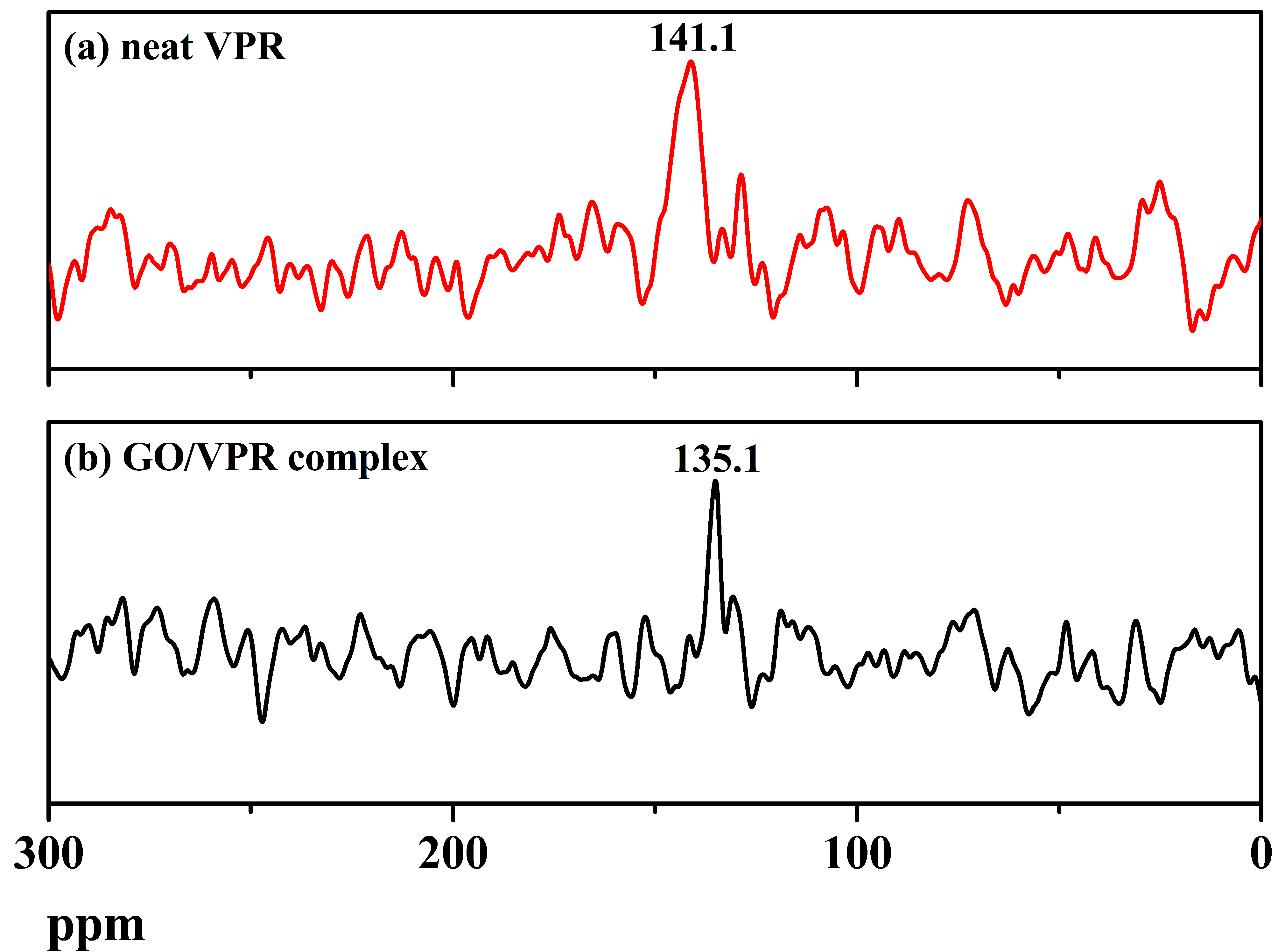


**Figure S8.** NMR spectra of neat VPR and GO/VPR complex.

**S5. Preparation of GO/SBR composites without VPR**

**
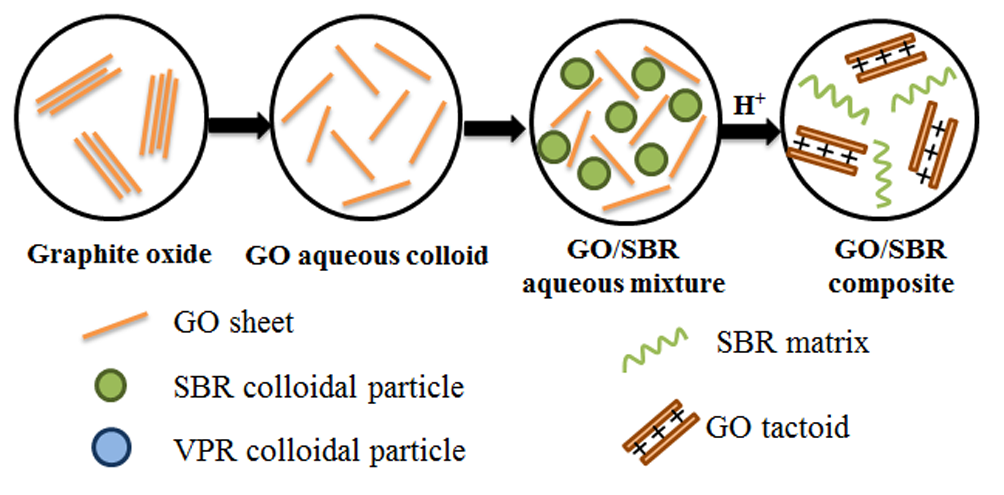
**

**Figure S9.** Schematic diagram of preparation process of GO/SBR composite without VPR

**
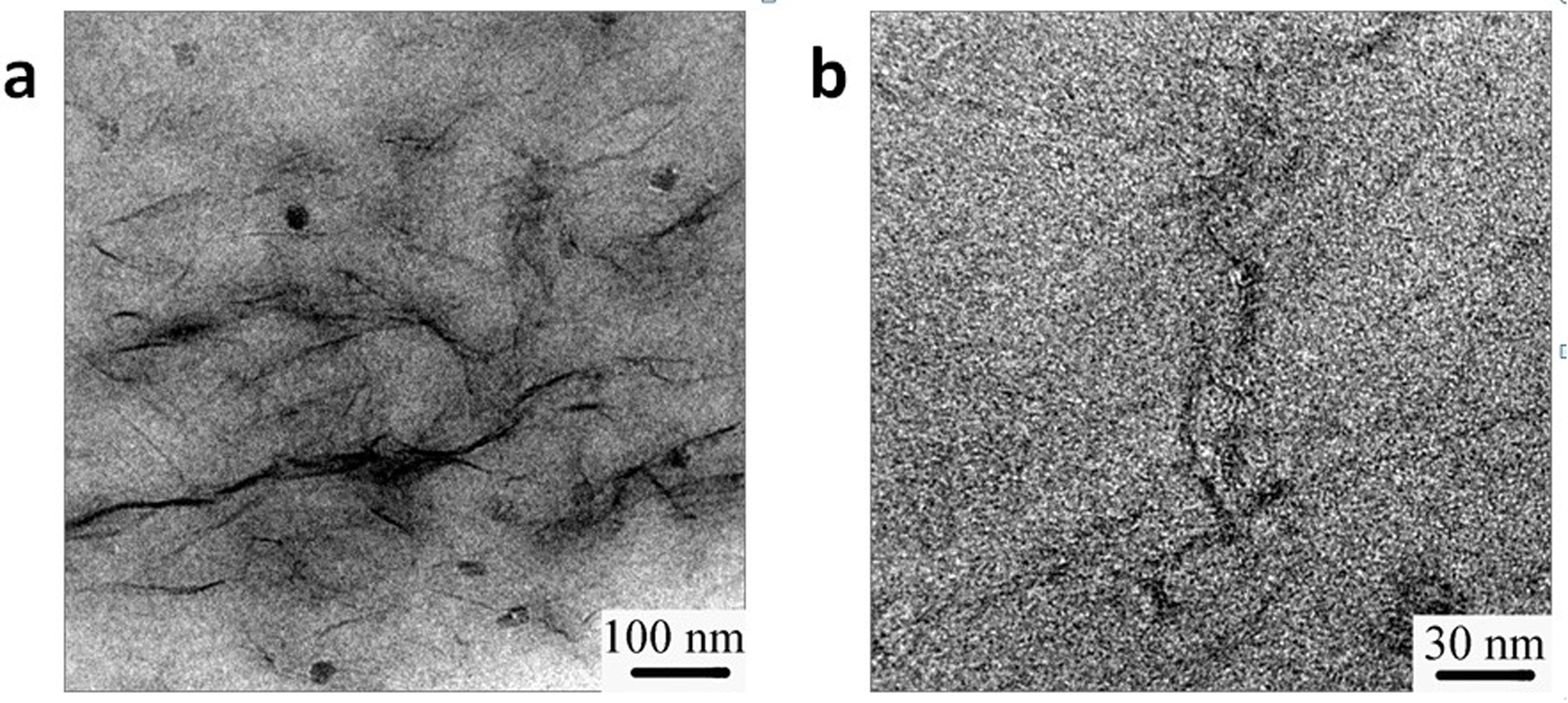
**

**Figure S10.** TEM images of GO/SBR composite (2.0 vol.% GO) without VPR.

**S6 Mechanical properties of** **GO/SBR composite**


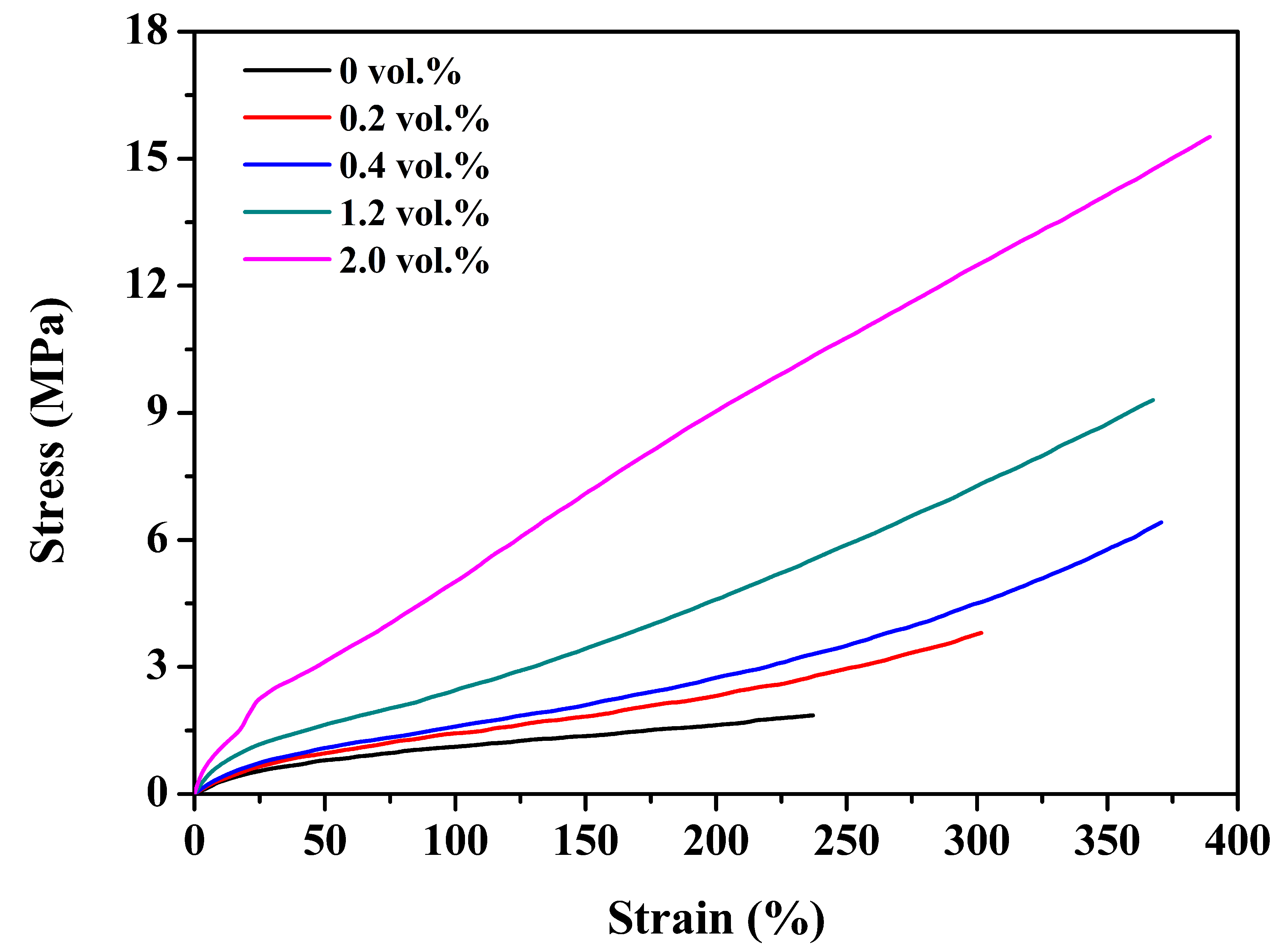


**Figure S11.** Stress–strain behavior of the GO/SBR composite at different GO loadings.

| **Table S1. Mechanical properties of GO/SBR composites** | | | | | |
| --- | --- | --- | --- | --- | --- |
| **GO volume fraction (vol.%)** | 0 | 0.2 | 0.4 | 1.2 | 2.0 |
| **Density (g/cm^3^)** | 1.00 | 1.01 | 1.02 | 1.02 | 1.03 |
| **Hardness (Shore A)** | 52 | 56 | 60 | 68 | 78 |
| **Tensile strength (MPa)** | 1.9 | 3.7 | 6.6 | 9.5 | 16.1 |
| **Elongation at break (%)** | 242 | 306 | 391 | 374 | 395 |
| **Modulus at 100% (MPa)** | 1.1 | 1.4 | 1.6 | 2.5 | 5.2 |
| **Modulus at 300% (MPa)** | -- | 3.6 | 4.4 | 7.4 | 13.0 |
| **Tear strength (kN/m)** | 18.1 | 22.5 | 35.2 | 47.0 | 49.7 |

**S7 Mechanical properties of SBR composites filled with various fillers**

| **Table S2. Mechanical properties of SBR composites filled with different fillers** | | | | | | | | |
| --- | --- | --- | --- | --- | --- | --- | --- | --- |
| **Filler type** | **Filler volume fraction (vol.%)** | **Density**  **(g/cm^3^)** | **Hardness**  **（Shore A）** | **Tensile strength (MPa)** | **Elongation at break (%)** | **Modulus at 100%**  **(MPa)** | **Modulus at 300%**  **(MPa)** | **Tear strength**  **(kN/m)** |
| **GO** | 2.0 | 1.03 | 78 | 16.1 | 395 | 5.2 | 13.0 | 49.8 |
| **N115** | 13.1 | 1.13 | 69 | 19.6 | 421 | 2.8 | 11.5 | 31.4 |
| **N220** | 13.1 | 1.13 | 68 | 16.6 | 303 | 3.2 | - | 24.2 |
| **N234** | 13.1 | 1.13 | 68 | 19.9 | 421 | 2.7 | 12.3 | 30.8 |
| **N326** | 13.1 | 1.13 | 67 | 18.2 | 384 | 2.6 | 12.4 | 18.5 |
| **N330** | 13.1 | 1.13 | 66 | 15.9 | 373 | 2.6 | 11.9 | 25.3 |
| **N375** | 13.1 | 1.13 | 69 | 17.0 | 332 | 3.0 | 14.5 | 29.0 |
| **N539** | 16.7 | 1.13 | 67 | 14.6 | 346 | 3.2 | 12.8 | 25.3 |
| **N550** | 16.7 | 1.13 | 69 | 18.4 | 336 | 3.7 | 16.4 | 22.0 |
| **N774** | 16.7 | 1.13 | 67 | 16.7 | 378 | 2.8 | 12.0 | 23.2 |
| **N990** | 20.0 | 1.14 | 65 | 7.8 | 285 | 2.6 | - | 15.9 |

**S8 Quantitative analysis of GO sheet thickness in GO/SBR composite**

According to the Nielsen model[^1^](#_ENREF_1), the relationship between the permeability of the filled polymer and that of the unfilled polymer is

(1)

where P_f_ is the permeability of the filled polymer, *P_u_* is the permeability of the unfilled polymer, φ_u_ is the volume fraction of polymer and τ is the shape factor.

Zhang[^2^](#_ENREF_2) amended the Nielsen model, establishing a relation between the thickness of the layered filler and permeability:

(2)

where *ϕ* is the volume fraction of filler (*ϕ* = 1− *ϕ* _u_), *r* is the radius of the layered filler, and *d*_0_ is the thickness of the layered filler.

According to equation (2) and a permeability value (P_u_=6.6, P_f_=2.32), the thickness of an individual GO-based sheet dispersed in the GO/SBR composite with 2.0 vol.% of GO was calculated to be *d*_0_ = 1.47 nm.

**S9 Properties of silica/SBR composite and GO-silica/SBR composite**

| **Table S3. Mechanical properties of silica/SBR composite and GO-silica/SBR composite** | | |
| --- | --- | --- |
| **Samples** | Silica/SBR composite | GO-silica/SBR composite |
| **Density (g/cm^3^)** | 1.16 | 1.16 |
| **Hardness (Shore A)** | 73 | 82 |
| **Tensile strength (MPa)** | 21.5 | 23.8 |
| **Elongation at break (%)** | 594 | 633 |
| **Modulus at 100% (MPa)** | 2.1 | 2.6 |
| **Modulus at 300% (MPa)** | 7.2 | 8.3 |
| **Tear strength (kN/m)** | 53.9 | 60.4 |

| **Table S4. Dynamic properties of silica/SBR composite and GO-silica/SBR composite** | | |
| --- | --- | --- |
| **Samples** | Silica/SBR composite | GO-silica/SBR composite |
| **Tan δ (60 ^o^C）** | 0.12 | 0.10 |
| **Akron abrasion loss (cm^3^/1.61km)** | 0.27 | 0.19 |

**S10 GO/SBR composite prepared by direct mechanical blending method**


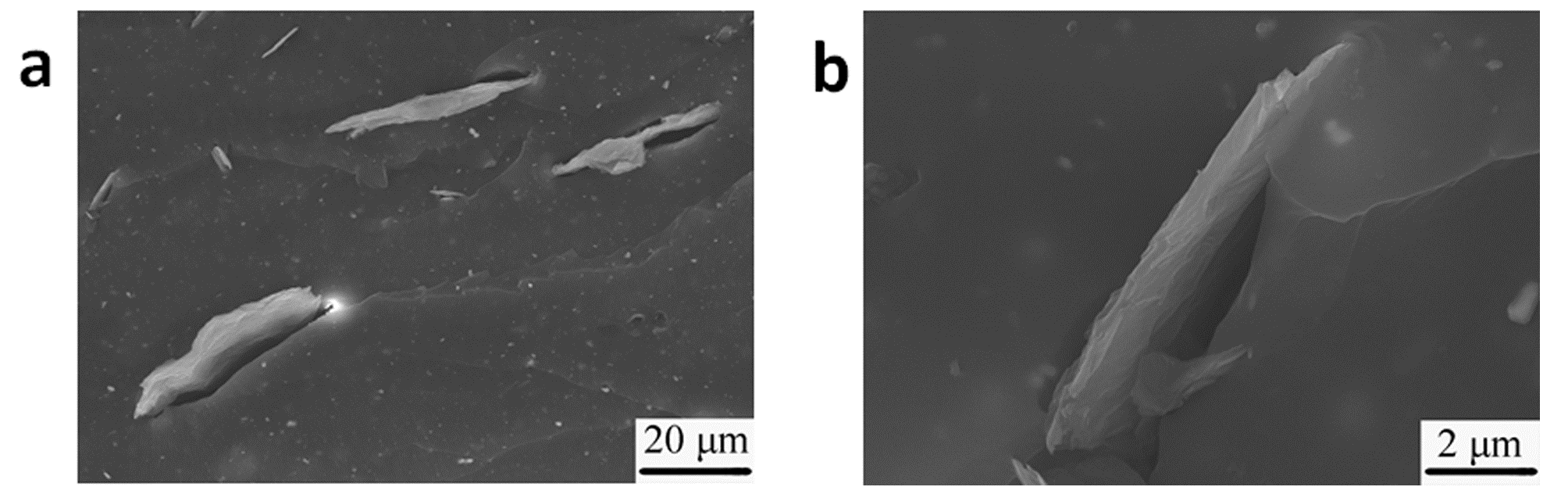


**Figure S12.** SEM images (a, 1000x magnification; b, 10000x magnification) of GO/SBR composite with 2.0 vol.% GO prepared by direct mechanical blending method.

| **Table S5. Mechanical properties of SBR/GO(2.0vol%) composite prepared by direct mechanical blending method** | |
| --- | --- |
| **Samples** | GO/SBR composite |
| **Hardness (Shore A)** | 55 |
| **Tensile strength (MPa)** | 1.9 |
| **Elongation at break (%)** | 190 |
| **Modulus at 100% (MPa)** | 1.4 |
| **Modulus at 300% (MPa)** | -- |
| **Tear strength (kN/m)** | 18.1 |

**References**

1. Lewis, T. & Nielsen, L. Dynamic mechanical properties of particulate-filled composites. *J. Appl. Polym. Sci.* **14,** 1449-1471 (1970).

2. Zhang, H. F. Structure and Properties of Layered Silicate/Rubber Nanocomposites. Beijing University of Chemical Technology Beijing, China Doctor Dissertation (2004).
